# Supplementary material for: Cues to gender and racial identity reduce creativity in diverse social networks
Source: Sci Rep. 2021 May 13;11:10261. doi: 10.1038/s41598-021-89498-5 (PMC8119436; doi:10.1038/s41598-021-89498-5)
Supplement: Supplementary file 1 — Supplementary Information 1 [file 41598_2021_89498_MOESM1_ESM.pdf]

# Supplementary Information for

## Cues to gender and racial identity reduce creativity in diverse social networks

**Raiyan Abdul Baten, Richard Aslin, Gourab Ghoshal, and Ehsan Hoque**

**Corresponding Author: Ehsan Hoque**
**E-mail: mehoque@cs.rochester.edu**

### This PDF file includes:

Supplementary text
Figs. S1 to S4
Tables S1 to S10
SI References

## Supporting Information Text

**Capturing Inter-ego Semantic Similarity: Statistical Test Details.** From each round, we collected pairs of egos who shared (a) 2 common alters (i.e., exactly the same stimuli), (b) 1 common alter and (c) no common alter. Within these subgroups, we computed the semantic similarities between every ego-pair's stimulated ideas in turn-2.

We adopted a $3 \times 2$ factorial design to analyze the data, with 3 levels in the number of common alters (i.e., the a, b and c subgroups above) and 2 levels in the study condition factor (i.e., control and treatment). In doing so, we employed the Aligned Rank Transform (ART) procedure (1), which is a linear mixed model-based non-parametric test. Figure 3 in the main manuscript visualizes the results. We found significant main effects for both of the factors (Number of common alters: $F(2, 25964) = 135.94$, $P < 10^{-15}$; Study condition: $F(1, 25964) = 369.98$, $P < 10^{-15}$). We also found a significant interaction between the two factors ($F(2, 25964) = 17.81$, $P < 10^{-7}$).

Post-hoc analysis on the ART-fitted model revealed that the semantic similarity between ego-pairs increases as their number of common alters increases (0 vs 1 common alter: $t(25964) = -10.18$, $P < 10^{-4}$; 1 vs 2 common alter(s): $t(25964) = -9.01$, $P < 10^{-4}$). Further pairwise comparisons using 2-tailed tests showed that this trend holds individually in both of the control and treatment conditions. In the control condition, the inter-ego similarities increased significantly as the number of common alters increased from 0 to 1 and also from 1 to 2 (0 vs. 1 common alter: $t(25964) = -10.61$, $P < 10^{-4}$; 1 vs. 2 common alters: $t(25964) = -8.55$, $P < 10^{-4}$). The same held for the treatment condition (0 vs. 1 common alter: $t(25964) = -4.71$, $P < 10^{-4}$; 1 vs. 2 common alters: $t(25964) = -5.15$, $P < 10^{-4}$). These trends intuitively follow the argument that inter-follower similarities can stem from having common stimulation sources.

Notably, we observed that the inter-ego semantic similarities are significantly higher in the treatment condition compared to their control counterparts, as revealed by post-hoc analysis on the study condition factor in the ART-fitted model ($t(25964) = 19.24$, $P < 10^{-4}$). Further pairwise comparisons using 2-tailed tests revealed that this result holds for all of the three common-alter-based subgroups (treatment vs. control; 2 common alters: $t(25964) = 5.27$, $P < 10^{-4}$; 1 common alter: $t(25964) = 13.79$, $P < 10^{-4}$; 0 common alter: $t(25964) = 17.87$, $P < 10^{-4}$). In addition, we observed that with time, the inter-ego semantic similarities in both of the control and treatment conditions show increasing trends (Pearson's correlation, $r$=0.13, $P < 10^{-4}$ in the treatment condition; $r$=0.12, $P < 10^{-4}$ in the control condition). All of the $P$-values reported here have been corrected for multiple comparisons using Holm's sequential Bonferroni procedure whenever needed. SI Tables S5-S10 capture these results.

## Supplementary Figures and Tables

The supplementary figures and tables are listed below.

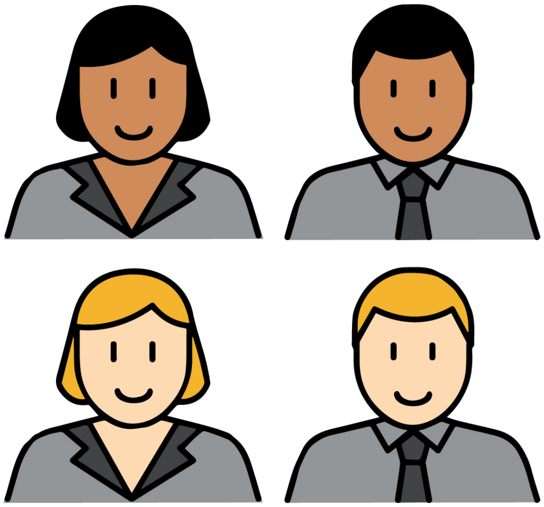

**Fig. S1.** Avatars used for depicting demographic information. Top row: Non-White female and Non-White male; Bottom row: White female and White male.

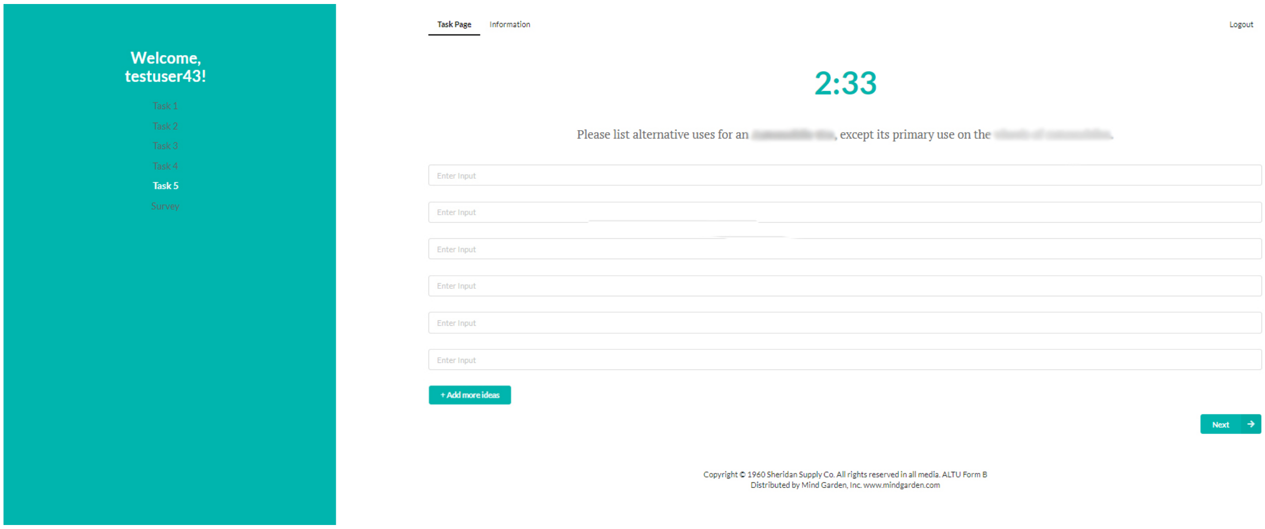


**Fig. S2.** Study interface: Initial (turn-1) idea submission interface for egos in both of the study conditions. This interface is used for recording the alters' ideas as well.

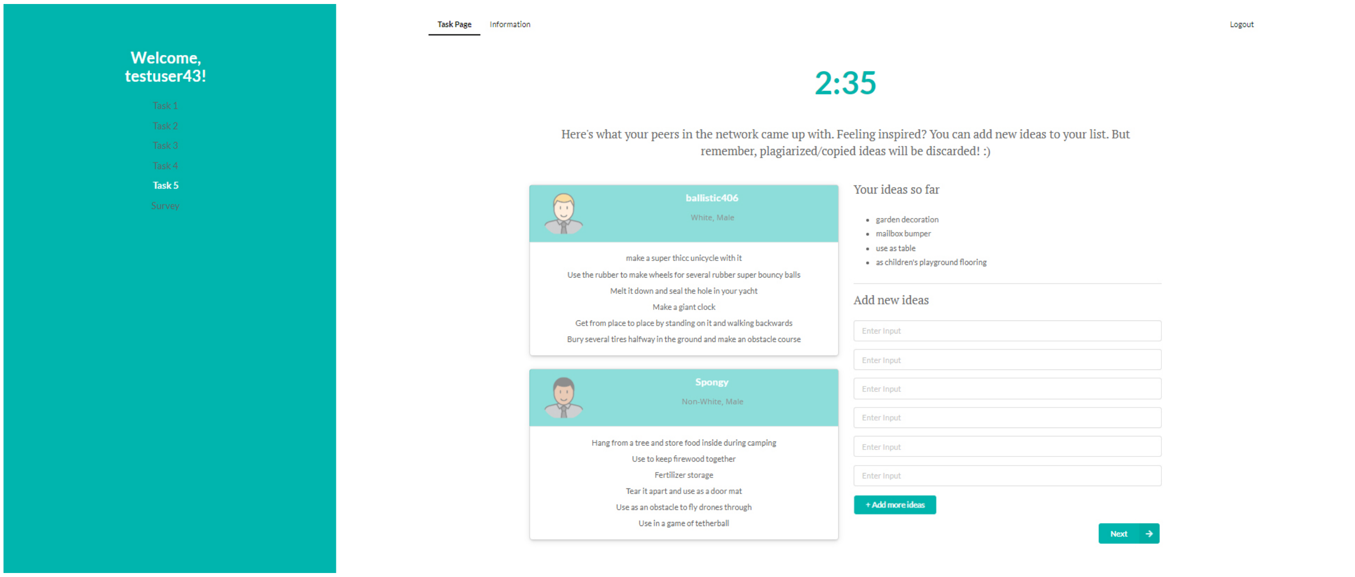


**Fig. S3.** Study interface: Turn-2 interface for the egos in the treatment condition. The alters' ideas are shown on the left-side cards. In the control condition, only the usernames and ideas of the alters are shown.

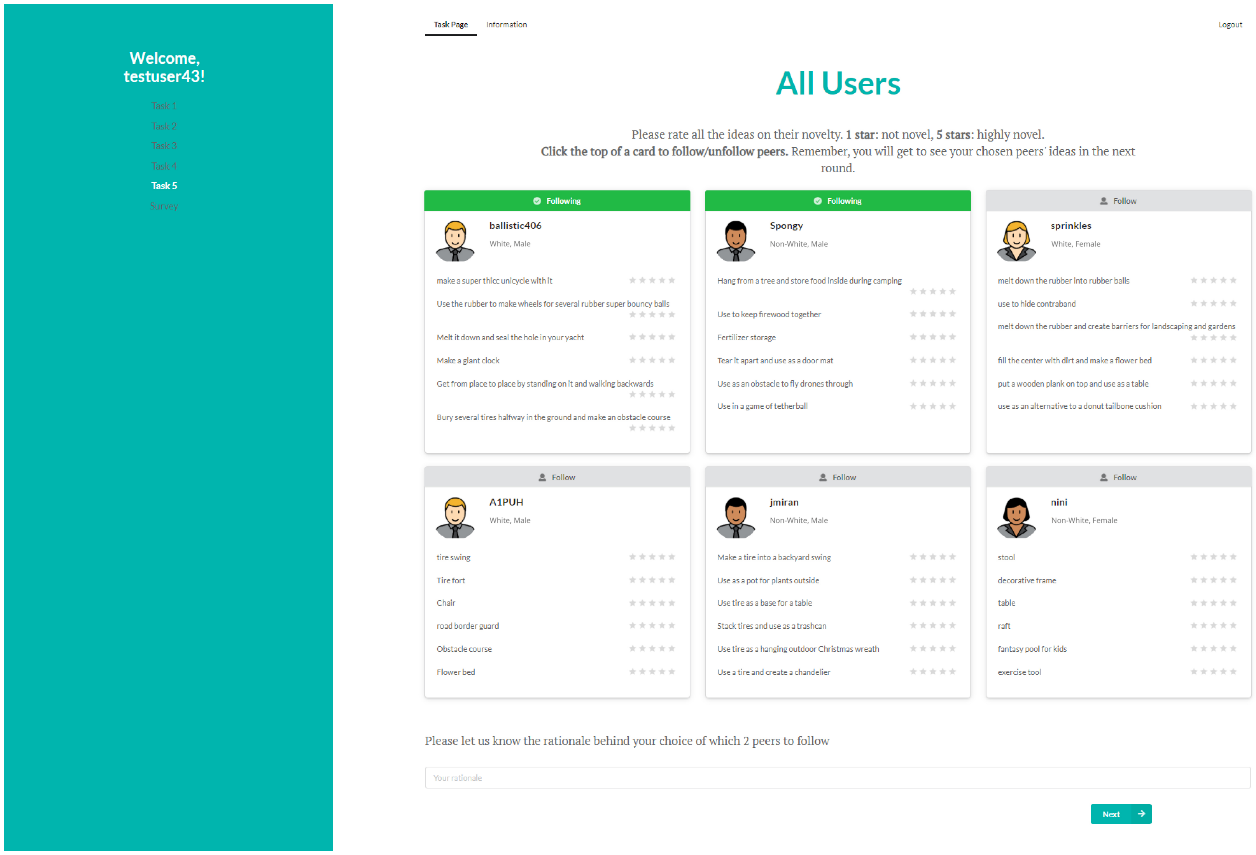


**Fig. S4.** Study interface: Rating and rewiring interface for the egos in the treatment condition. In the control condition, only the usernames and ideas of the alters are shown.

**Table S1. Link formation dynamics in the control condition. Summary results from the Monte Carlo Maximum Likelihood Estimation fit in the STERGM model.** ***$P < 0.001$

| | $\beta$ | Std. Error | $Z$ value | Pr($> \|Z\|$) | |
|---|---|---|---|---|---|
| Edges | $-4.496$ | $0.295$ | $-15.220$ | $< 1e-04$ | *** |
| Alters' non-redundant idea count | $0.324$ | $0.059$ | $5.505$ | $< 1e-04$ | *** |
| Gender-based homophily | $-0.014$ | $0.145$ | $-0.094$ | $0.925$ | |
| Race-based homophily | $0.056$ | $0.144$ | $0.389$ | $0.698$ | |

**Table S2. Link persistence dynamics in the control condition. Summary results from the Monte Carlo Maximum Likelihood Estimation fit in the STERGM model.** ***$P < 0.001$, *$P < 0.05$

| | $\beta$ | Std. Error | $Z$ value | Pr($>|Z|$) | |
|---|---|---|---|---|---|
| Edges | $-0.577$ | $0.290$ | $-1.989$ | $0.0467$ | * |
| Alters' non-redundant idea count | $0.417$ | $0.060$ | $6.952$ | $< 1e-04$ | *** |
| Gender-based homophily | $-0.202$ | $0.167$ | $-1.207$ | $0.2275$ | |
| Race-based homophily | $0.179$ | $0.166$ | $1.077$ | $0.2815$ | |

**Table S3. Link formation dynamics in the treatment condition. Summary results from the Monte Carlo Maximum Likelihood Estimation fit in the STERGM model.** ***$P < 0.001$

| | $\beta$ | Std. Error | $Z$ value | Pr($> \|Z\|$) | |
|---|---|---|---|---|---|
| Edges | $-3.740$ | $0.246$ | $-15.230$ | $< 1e - 04$ | *** |
| Alters' non-redundant idea count | $0.197$ | $0.050$ | $3.933$ | $< 1e - 04$ | *** |
| Gender-based homophily | $-0.117$ | $0.134$ | $-0.879$ | $0.379$ | |
| Race-based homophily | $0.096$ | $0.134$ | $0.714$ | $0.475$ | |

**Table S4. Link persistence dynamics in the treatment condition. Summary results from the Monte Carlo Maximum Likelihood Estimation fit in the STERGM model.** ***$P < 0.001$, **$P < 0.01$

| | $\beta$ | Std. Error | $Z$ value | Pr($> \|Z\|$) | |
|---|---|---|---|---|---|
| Edges | $-0.751$ | $0.290$ | $-2.588$ | $0.0097$ | ** |
| Alters' non-redundant idea count | $0.355$ | $0.058$ | $6.075$ | $< 1e-04$ | *** |
| Gender-based homophily | $0.599$ | $0.160$ | $3.743$ | $0.0002$ | *** |
| Race-based homophily | $-0.067$ | $0.158$ | $-0.425$ | $0.671$ | |

**Table S5. Omnibus test results for analyzing the inter-ego semantic similarities under various conditions. The cosine similarity between idea-sets of pairs of egos is the response variable. The analysis of variance of Aligned Rank Transformed data is run on a model with two factors: the number of popular alters of the egos ($3$ levels) and the study condition ($2$ levels). The degrees of freedom are specified using the Kenward-Roger method.**

| | Df | Df.res | $F$ | Pr($> F$) |
|---|---|---|---|---|
| Number of Popular Alters | $2$ | $25964$ | $135.944$ | $< 2.22e-16$ |
| Condition | $1$ | $25964$ | $369.983$ | $< 2.22e-16$ |
| NumPopularAlters:Condition | $2$ | $25964$ | $17.811$ | $1.86e-8$ |

**Table S6. Post-hoc contrast analysis among the three levels in the 'number of popular alters' factor from the fitted model reported in Table S5. The degrees of freedom are specified using the Kenward-Roger method. The $P$-values are adjusted using Holm's sequential Bonferroni procedure.**

| Contrast | SE | df | $t$ | $p$ |
|---|---|---|---|---|
| 0 common alter-1 common alter | $102$ | $25964$ | $-10.176$ | $< 0.0001$ |
| 0 common alter-2 common alters | $141$ | $25964$ | $-15.999$ | $< 0.0001$ |
| 1 common alter-2 common alters | $136$ | $25964$ | $-9.014$ | $< 0.0001$ |

**Table S7. Post-hoc contrast analysis among the two levels in the 'study condition' factor from the fitted model reported in Table S5. The degree of freedom is specified using the Kenward-Roger method. The $P$-value is adjusted using Holm's sequential Bonferroni procedure.**

| Contrast | SE | df | $t$ | $p$ |
|---|---|---|---|---|
| control-treatment | $104$ | $25964$ | $-19.235$ | $< 0.0001$ |

**Table S8. Comparisons of cosine similarities among the three levels of 'number of popular alters' factor in the control condition. $2$-tailed tests. The $P$-values are adjusted using Holm's sequential Bonferroni procedure.**

| Contrast | SE | df | $t$ | $P$ |
|---|---|---|---|---|
| 0 common alter-1 common alter | $0.00225$ | $25964$ | $-10.614$ | $< 0.0001$ |
| 0 common alter-2 common alters | $0.00302$ | $25964$ | $-16.067$ | $< 0.0001$ |
| 1 common alter-2 common alters | $0.00288$ | $25964$ | $-8.551$ | $< 0.0001$ |

**Table S9. Comparisons of cosine similarities among the three levels of 'number of popular alters' factor in the treatment condition. $2$-tailed tests. The $P$-values are adjusted using Holm's sequential Bonferroni procedure.**

| Contrast | SE | df | $t$ | $P$ |
|---|---|---|---|---|
| 0 common alter-1 common alter | $0.00216$ | $25964$ | $-4.709$ | $< 0.0001$ |
| 0 common alter-2 common alters | $0.00312$ | $25964$ | $-8.274$ | $< 0.0001$ |
| 1 common alter-2 common alters | $0.00304$ | $25964$ | $-5.152$ | $< 0.0001$ |

**Table S10. Comparisons of cosine similarities between the two study conditions. $2$-tailed tests. The $P$-values are adjusted using Holm's sequential Bonferroni procedure.**

| Contrast | SE | df | $t$ | $P$ |
|---|---|---|---|---|
| control-treatment; 2 common alters | $0.00365$ | $25964$ | $-5.271$ | $< 0.0001$ |
| control-treatment; 1 common alter | $0.00205$ | $25964$ | $-13.786$ | $< 0.0001$ |
| control-treatment; 0 common alter | $0.00235$ | $25964$ | $-17.865$ | $< 0.0001$ |

## References

1. JO Wobbrock, L Findlater, D Gergle, JJ Higgins, The Aligned Rank Transform for nonparametric factorial analyses using only ANOVA procedures in *Proceedings of the SIGCHI Conference on Human Factors in Computing Systems.* pp. 143–146 (2011).
